# The celebrity effect on gaze following in older and young adults

**DOI:** 10.1186/s41155-024-00319-w

**Published:** 2024-08-27

**Authors:** Airui Chen, Zhaojun Yuan, Sihan Zhou, Qingqing Yu, Fangyuan Zhang, Bo Dong

**Affiliations:** Department of Psychology, Suzhou University of Science and Technology, Xuefu Road 99, Suzhou, 215009 China

**Keywords:** Gaze following, Celebrity effect, Gaze cue effect, Older adults, Young adults

## Abstract

**Background:**

In daily life, people often follow others’ gaze direction to infer their attention and mental state. This phenomenon is known as gaze following.

**Objective:**

This study aimed to explore whether gaze following in different age groups is influenced by celebrity identity.

**Methods:**

We recruited 70 participants, including 35 older adults and 35 young adults. The experimental materials consisted of three faces with different identity information (a political leader, a movie star, and an ordinary person). Each face had left and right gaze conditions. Targets and cues were presented with both longer and shorter stimulus onset asynchrony (SOA) conditions.

**Results:**

Both older adults and young adults exhibited similar gaze following behaviors. Importantly, the celebrity effect on gaze following was observed in both groups, with stronger effects induced by the leader’s and star’s gazes compared to the ordinary person’s gaze. Older adults showed a larger facilitation effect under the longer SOA condition compared to the shorter SOA, while no such SOA-related facilitation effect was found for young adults.

**Conclusion:**

These findings suggest that older adults can integrate social information from others’ faces (celebrity identity) into the process of gaze following as effectively as young adults.

## Introduction

Gaze following is the phenomenon where individuals automatically redirect their attention according to another’s gaze direction to speculate on the other’s attentional object and intention. (Moore et al., (Moore et al. 2014)). Many researchers are interested in this phenomenon (Driver IV et al., (Driver et al. 1999); Friesen & Kingstone, (Friesen and Kingstone 1998); Friesen et al., (Friesen et al. 2004); Frischen et al., (Frischen et al. 2007); Langton & Bruce, (Langton and Bruce 1999); Wang et al., (Wang et al. 2020)) and employ the cue-target paradigm to investigate gaze following. In this paradigm, a left-gazed or right-gazed face is initially presented as a cue in the center of the screen, followed by a target appearing on either the cued or uncued side. Reaction time under cued and uncued conditions is subtracted to derive the gaze following indicator, known as the gazing cue effect (GCE). The greater the effects are, the stronger the gaze following is.

Social information can be integrated into the gaze following process. Specifically, gaze following is influenced by social context evaluation, the observer’s knowledge, and cultural factor (Dalmaso et al., (Dalmaso et al. 2012); Jones et al., (Jones et al. 2010)). Observers exhibit more significant gaze following when presented with dominant facial cues (Jones et al., (Jones et al. 2010)). Under the Western social and cultural background, white individuals tend to inhibit gaze following when observing black individuals (Dalmaso et al. 2012), (Dalmaso et al. 2015); Pavan et al., (Pavan et al. 2011); Weisbuch et al., (Weisbuch et al. 2017); Zhang et al., (Zhang et al. 2021)). For Asian participants, gaze cues from white faces are stronger than those from Asian faces (Chen & Zhao, (Chen and Zhao 2015); Zhang et al., (Zhang et al. 2021)). These findings support the view that top-down factors or social information can influence gaze following (Dalmaso et al., (Dalmaso et al. 2020)).

However, most participants in the aforementioned studies were young adults. Thus, for older adults, is gaze following influenced by social information as it is for young people? There are two potential scenarios. First, compared to young adults, with the weakening connection of the attention network, older adults have fewer cognitive resources (Lien et al., (Lien et al. 2011)); their speed of processing information is slower (Bennett et al., (Bennett et al. 2004); Salthouse, (Salthouse 1996)); and also, the cognitive control flexibility is decreasing. It is difficult for them to direct their attention according to different cognitive contexts (Amenedo Losada et al., (Amenedo 2012); Madden et al., (Madden et al. 2014)). Additionally, some studies have found that the ability to follow others’ gaze direction decreases with age (Mason, (Mason 1986); McKay et al., (McKay et al. 2021); Slessor et al., (Slessor et al. 2008), (Slessor et al. 2010), (Slessor et al. 2016)). Consequently, older adults may struggle to integrate social information into gaze following due to cognitive aging. This perspective is supported by research indicating that the age of the face modulates gaze following only in young adults, not in older adults (Ciardo et al., (Ciardo et al. 2014)). Conversely, other studies have found that older adults can follow others’ gaze direction as effectively as young adults (Deroche et al., (Deroche et al. 2016); Fernandes et al., (Fernandes et al. 2024); Gayzur et al., (Gayzur et al. 2014)), suggesting they might integrate social information into gaze following similarly to younger individuals. Given these conflicting findings, it remains unclear whether gaze following in older adults is modulated by social information as it is in young adults.

Celebrity worship refers to an individual’s emotional attachment and social identification with a person they admire (Brooks, (Brooks 2021)). The core elements of this phenomenon are psychological identification and emotional attachment. Leaders and stars are significant types of celebrities. Previous research indicates that individuals with higher social status tend to elicit stronger gaze following behaviors, particularly among young people (Capozzi et al., (Capozzi et al. 2016); Dalmaso et al., (Dalmaso et al. 2012), (Dalmaso et al. 2014)). Therefore, we predict that leaders and celebrities will elicit stronger gaze following responses compared to ordinary individuals among young people. However, for older adults, it remains uncertain whether their gaze following is influenced by the celebrity status of faces. Furthermore, if such an influence exists, it is unclear whether older adults will exhibit the same trends as younger individuals.

In this study, we compared the gaze cue effect of the star and the leader in both young and older adults. We selected face photos of Zedong Mao and Jackie Chan as face models for two reasons. First, both the older and the young participants are very familiar with these two types of celebrities. Second, these two figures are highly representative of the political leader and the star, respectively. We investigated the modulation effect of celebrity faces on gaze following over short and long temporal courses, given that stimulus onset asynchrony (SOA) between the face cue and target is a critical factor in gaze following (Friesen & Kingstone, (Friesen and Kingstone 1998)). Additionally, the time window in which young and older adults are influenced by the celebrity effect may differ. This study included three independent factors: face type, gaze cue validity, and SOA (stimulus onset asynchrony) (200 ms and 600 ms). If celebrity identity affects gaze following, the gazing cue effects will vary depending on the face types. Otherwise, there will be no significant differences in the magnitude of gaze following under these conditions.

### Method

#### Participants

There are 70 participants. They are 35 older adults (16 males and 19 females) aged from 65 to 75 years old (*M* = 68.53, *SE* = 1.78) and 35 young adults (15 males and 20 females) aged from 18 to 22 years old (*M* = 20.2, *SE*= 0.76) participated in the experiment. All of them were right-handed and healthy. The sample size was determined based on previous relevant studies (Hietanen et al., (Hietanen et al. 2006); Ji et al., (Ji et al. 2022); Shi et al., (Shi et al. 2010)), and a two-tailed power analysis using G*Power (Version 3.1.9.4) (Faul et al., (Faul et al. 2007)) confirmed that a sample size of 28 participants in each age range would achieve 80% power to detect an attentional effect induced by social cues (Cohen’s*d*= 0.8, which is an average effect size found in previous studies (see Hietanen et al., (Hietanen et al. 2006); Ji et al., (Ji et al. 2022); Shi et al., (Shi et al. 2010)). All participants had normal or corrected visual acuity and no color blindness or color weakness. The participants were paid after the experiment. This study was conducted in accordance with the Declaration of Helsinki and approved by the ethical committee of Suzhou University of Science and Technology.

#### Design

This experiment was a 3 (face type) × 2 (cue validity) × 2 (SOA) × 2 (age) mixed design. The between-subject variable was age. The three face types included a political leader, a movie star, and an ordinary person. Cue validity comprised neutral, valid, and invalid conditions. If the gaze direction of the model matched the location of the stimulus “★,” the gaze cue was valid; if the gaze direction was opposite to the stimulus location, it was invalid; if the model gazed directly ahead, it was a neutral cue. There were two SOA conditions: 200 ms and 600 ms. The dependent variables were reaction time and accuracy.

#### Materials

The experimental program was written with E-Prime software and run on the LAPTOP-0BQLFES5 computer. The display was Intel (R) UHD Graphics 620, the resolution was 1920 × 1080, and the refresh rate was 60 HZ. The experiment used the IBM keyboard for collecting participants’ reactions.

The screen background was white (RGB, 255, 255, 255), with a central black fixation point “ + ” (0.7° × 0.7°). The size of each face was 3° × 4°, with an eye area of 0.8° × 0.2°. The target stimulus was a black “★” (0.7° × 0.7°), positioned 5.5° away from the center of the face. The political leader was represented by a photo of Zedong Mao, and the star by a photo of Jackie Chan, both with neutral expressions. The ordinary person was represented by a photo of a male with a neutral expression. Each face had four gaze directions: looking straight, looking left, looking right, and closing the eyes. Photoshop software was used to adjust the gaze directions and process 12 face pictures with consistent brightness (brightness, 174). All the images were in black and white. The three models were aged between 50 and 60 years to exclude the effects of the model’s age on the experiment.

#### Procedure

The experiment was conducted in a quiet room. The participants were asked to sit in front of the screen at a distance of 50 cm from the screen with their chin resting on a chin rest. Their responses were to press the F and G keys on a keyboard with the index and middle fingers in their dominant hand. Before the formal experiment, the participants were told to complete the practice experiment. Only those who achieved an accuracy of at least 80% in the practice session were allowed to proceed to the formal experiment.

**Fig. 1:**
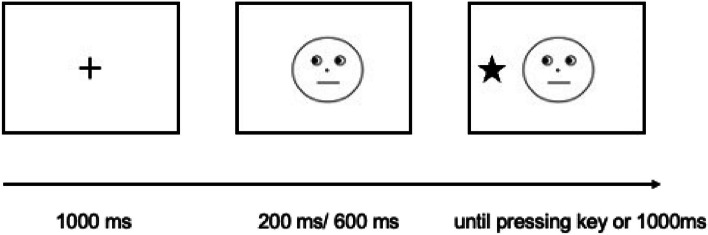
The procedure in the experiment

According to the previous studies in the research area of gaze following, we used the classic cue-target paradigm (Friesen & Kingstone, (Friesen and Kingstone 1998)). In the practice task, a fixation point was presented for 1000 ms in the center of the display screen. After the fixation disappeared, a face with a left gaze, right gaze, or closed gaze appeared. After an interval of 200 ms or 600 ms, the target stimulus “★” was presented on either the left or right side of the face until the participant pressed a key within 1000 ms. If the model’s eyes were closed (i.e., catch trial), participants were instructed not to press any key. Otherwise, they were to respond as quickly and accurately as possible according to the target position, pressing the F key for targets on the left and the G key for targets on the right (i.e., critical trial). The formal experiment followed the same procedure as the practice task (see Fig. 1). The practice task consisted of 60 trials, including 48 critical trials and 12 catch trials. The formal experiment comprised 480 trials, divided into 10 blocks, with 48 catch trials in total. All face photos were presented randomly. After the experiment, the participants’ familiarity with the three face photos was tested. All participants reported that they were very familiar with the political leader (Zedong Mao) and the star (Jackie Chan), and were unfamiliar with the ordinary person.

## Results

We evaluated the accuracy of critical trials (where participants were required to press a key) and catch trials (where no key press was required). In the analysis of critical trials for older participants, one individual with an accuracy rate of 83%, which was more than three standard deviations below the mean (*M* = 94.23%, *SD* = 6.41%), was excluded. Similarly, in the analysis of catch trials, another older participant was excluded due to an accuracy rate of 83.10%, which also exceeded three standard deviations below the mean (*M* = 97.12%, *SD* = 4.25%). Subsequently, data from the remaining 33 older participants were analyzed in detail. For the young participants, the accuracy rates in the analysis of critical trials were within three standard deviations (*M* = 99.03%, *SD* = 2.01%). However, in the analysis of catch trials, one participant with an accuracy rate of 81%—falling outside three standard deviations from the mean (M = 93.29%, *SD* = 4.52%)—was excluded. Data from the remaining 34 young participants were further analyzed. Consequently, a total of 67 participants were retained for the study, consisting of 33 older participants and 34 young participants.

### Accuracy

We conducted a mixed ANOVA to evaluate accuracy variations across three face types (political leader, star, ordinary person), two stimulus onset asynchronies (SOA, 200 ms, 600 ms), three cue types (valid, invalid, neutral), and two age groups (older, young). The analysis revealed a significant main effect of cue validity (*M* = 0.984, *SD* = 0.003), *F* (2, 64) = 8.057, *p* = 0.001, *η*^*2*^ = 0.201. Accuracy was significantly higher for valid cues (*M* = 0.987, *SD* = 0.003) compared to invalid cues (*M* = 0.981, *SD* = 0.004), *p* < 0.001, and to neutral cues (*M* = 0.984, *SD* = 0.004), *p* = 0.023. Furthermore, neutral cues resulted in significantly higher accuracy than invalid cues (*M* = 0.981, *SD* = 0.004),* p* = 0.038. Additionally, the effect of age was significant (*p* = 0.017), with older participants demonstrating lower accuracy (*M* = 0.976, *SD* = 0.005) than younger participants (*M* = 0.992, *SD* = 0.004). No other main effects or interactions were found to be significant.

#### Reaction time

We conducted a mixed ANOVA to analyze reaction time involving three face types (political leader, star, ordinary person), two stimulus onset asynchronies (SOA, 200 ms, 600 ms), three cue types (valid, invalid, neutral), and two age groups (older, young). There was a notable main effect of SOA, *F* (1, 65) = 251.469,* p* < 0.001, *η*^*2*^ = 0.795; reaction time was significantly shorter for the 600 ms SOA (*M* = 454.979, *SD* = 10.536) compared to the 200 ms SOA (*M* = 507.293, *SD* = 11.841). Additionally, the main effect of cue validity was significant, *F* (2, 64) = 15.474, *p* < 0.001,* η*^*2*^ = 0.326, indicating longer reaction time when targets were not aligned with gaze direction (*M* = 504.457, *SD* = 14.301) compared to neutral gaze direction (*M* = 473.958, *SD* = 10.420), *p* = 0.001 and valid gaze direction (*M* = 464.994, *SD* = 10.653), *p* < 0.001. Reaction time under neutral gaze was significant longer than valid gaze, *p* < 0.001.

Furthermore, the main effect of age was significant, *F* (1, 65) = 108.961, *p* < 0.001, *η*^*2*^ = 0.626. Older participants (*M* = 596.848, *SD* = 15.793) had significantly longer reaction time than younger participants (*M* = 365.425, *SD* = 15.559). The interaction between face type and cue validity was significant,* F* (4, 62) = 3.806, *p* = 0.008, *η*^*2*^ = 0.197. Under the valid cue condition, the ordinary people (*M* = 469.923, *SD* = 10.982) was significantly greater than political leader (*M* = 461.102, *SD* = 10.500), *p* = 0.010; the ordinary people (*M* = 469.923, *SD* = 10.982) was significantly greater than the star (*M* = 463.956, *SD* = 10.881), *p* = 0.028; the political leader and the star has no significant effect. Under the invalid condition, the political leader (*M* = 508.275, *SD* = 14.432) was significantly greater than the ordinary people (*M* = 501.118, *SD* = 14.187), *p* = 0.002; the political leader and star or the star and the ordinary people have no significant effect. Under the neutral condition, the ordinary people (*M* = 476.780, *SD* = 10.841) was significantly greater than the political leader (*M* = 475.614, *SD* = 10.284), *p* = 0.011; the ordinary people (*M* = 476.780, *SD* = 10.841) was greater than the star (*M* = 469.923, *SD* = 10.478), *p* = 0.017; the political leader and the star has no significant effect. The interaction between SOA and cue validity was significant, *F* (2, 64) = 5.948, *p* = 0.004, *η*^*2*^ = 0.157. For 200 ms SOA, reaction time for invalid cues (*M* = 529.588, *SD* = 15.264) was greater than for neutral cues (*M* = 498.812, *SD* = 10.874), *p* = 0.001 and valid cues (*M* = 493.479, *SD* = 11.178), *p* = 0.013; reaction time for neutral cues was significantly longer than valid cues, *p* = 0.013. For 600 ms SOA, invalid cues (*M* = 479.326, *SD* = 13.550) resulted in longer reaction time than neutral cues (*M* = 449.104, *SD* = 10.266), *p* = 0.002 and valid cues (*M* = 436.508, *SD* = 10.406), *p* < 0.001; neutral cues resulted in longer reaction times than, *p* < 0.001. Lastly, the interaction between SOA, cue validity, and age was significant, *F* (2, 64) = 6.536, *p* = 0.003, *η*^*2*^ = 0.170 (Table 1.). The other main effects and interactions were not significant. Then, we used the gaze cueing effect to reveal the complex interplay of three factors in cognitive processing.

**Table 1:**
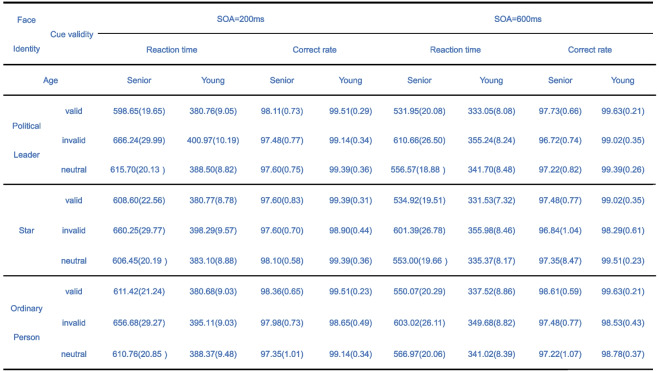
Average reaction time (ms) and accuracy (%) of correct reaction in the senior adults under various conditions

### Gaze cueing effect

Given that older participants exhibited an overall slower reaction time, compared to the younger participants, we calculated the gaze cueing effect (GCE) as a proportional value, following previous studies on gaze following (Morillo-Mendez et al., (Morillo-Mendez et al. 2023); Slessor et al., (Slessor et al. 2016)). This approach facilitates a more accurate comparison between older and younger participants. Additionally, to determine whether the processing of cues by older and younger adults reflected an attentional facilitation or inhibition, we compared responses to valid cue and invalid cue against neutral cue condition. The gaze cueing effect (GCE), facilitation effect (Faci), and incongruence effect (Incong) were calculated using the following formulas (Slessor et al., (Slessor et al. 2016)).

- GCE = (RT_incong – RT_cong) / RT_cong.
- Faci = (RT_neutral – RT_cong) / RT_cong.
- Incong = (RT_incong – RT_neutral) / RT_neutral.

**Fig. 2:**
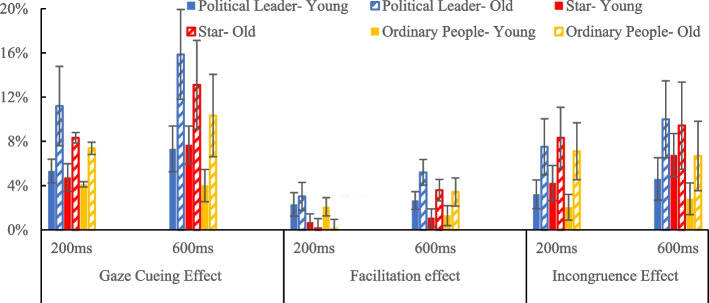
Gaze cueing effect, facilitation effect, and incongruence effect for each SOA, age, and face condition

We used a mixed ANOVA to analyze the gaze cueing effect (GCE) across three face types (political leader, star, ordinary person), two stimulus onset asynchronies (SOA, 200 ms, 600 ms), and two age groups (older, young). The main effect of SOA was significant, *F* (1, 65) = 12.844, *p* = 0.001, *η*^*2*^ = 0.165, with the 600 ms SOA (*M* = 0.097, *SD* = 0.020) showing a greater GCE than the 200 ms SOA (*M* = 0.068, *SD* = 0.016). In addition, the main effect of face type was significant, *F* (2, 64) = 7.411, *p* = 0.001, *η*^*2*^ = 0.188. The political leader (*M* = 0.10, *SD* = 0.02) elicited a greater GCE than the star (*M* = 0.085, *SD* = 0.02), *p* = 0.049, and the ordinary person (*M* = 0.07, *S*D = 0.02), *p* < 0.001, star (*M* = 0.085, *SD* = 0.02) also elicited a greater GCE than the ordinary person (*M* = 0.07, *SD* = 0.02), *p* = 0.018 (see Fig. 2). The other main effects and interactions were not significant.

### Facilitation effect

We used a mixed ANOVA to analyze the facilitation effect across three face types (political leader, star, ordinary person), two stimulus onset asynchronies (SOA, 200 ms, 600 ms), and two age groups (older, young). The main effect of face type was significant, *F* (2, 64) = 4.291, *p* = 0.018, *η*^*2*^ = 0.118. The political leader (*M* = 0.033, *SD* = 0.006) elicited a greater facilitation effect than the star (*M* = 0.014, *SD* = 0.005), *p* = 0.008, and the ordinary person (*M* = 0.017, *SD* = 0.006), *p* = 0.036. However, there was no significant difference between the star and ordinary person face types. Additionally, the main effect of SOA was significant, *F* (1, 65) = 11.818, *p* = 0.001, *η*^*2*^ = 0.154, with the 600 ms SOA (*M* = 0.029, *SD* = 0.004) resulting in a greater facilitation effect than the 200 ms SOA (*M* = 0.014, *SD* = 0.004). Furthermore, the interaction between SOA and age was significant, *F* (1, 65) = 12.018, *p* = 0.001, *η*^*2*^ = 0.156. For older participants, the 600 ms SOA (*M* = 0.041, *SD* = 0.006) produced a greater facilitation effect than the 200 ms SOA (*M* = 0.011, *SD* = 0.006), *p* = 0.006, while no significant difference was observed among young participants (see Fig. 2). At the 600 ms SOA, older participants (*M* = 0.041, *SD* = 0.006) exhibited a significantly greater facilitation effect than young participants (*M* = 0.017, *S*D = 0.005), *p* = 0.006. At the 200 ms SOA, there was no significant difference between older and young participants. The other main effects and interactions were not significant.

### Incongruence effect

We used a mixed ANOVA to analyze the incongruence effect across three face types (political leader, star, ordinary person), two stimulus onset asynchronies (SOA, 200 ms, 600 ms), and two age groups (older, young). The main effect of face type was significant, *F* (2, 64) = 3.610, *p* = 0.033, *η*^*2*^ = 0.101. The political leader (*M* = 0.063, *SD* = 0.016) elicited a greater incongruence effect than the ordinary person (*M* = 0.047,* SD* = 0.015), *p* = 0.031. The star (*M* = 0.072, *SD* = 0.015) also elicited a greater incongruence effect than the ordinary person, *p* = 0.010 (Table 2). However, there was no significant difference between the political leader and star face types (see Fig. 2).

**Table 2:**
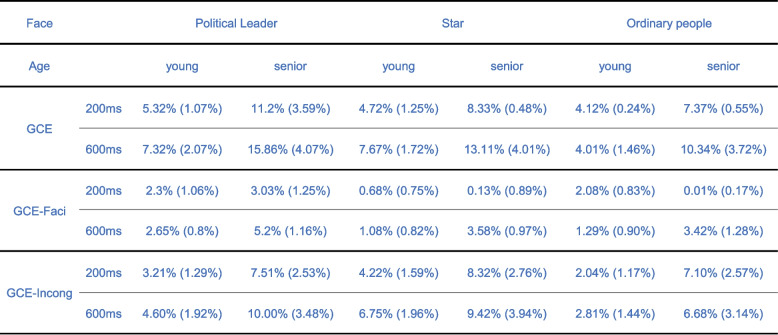
GCE, GCE-Faci, and GCE-Incong effects among young and older participants

## Discussion

This study employed a cue-target paradigm to investigate the celebrity effect on gaze following in older and younger adults. Both older and younger participants exhibited similar gaze following and celebrity effects. The gaze following effect under the political leader and star conditions was significantly greater than that under the ordinary person condition, indicating that social information, such as celebrity identity, can influence gaze following in both age groups. Importantly, the celebrity effect was observed in both the facilitation effect and the incongruence effect, through comparisons of neutral cues with valid and invalid cues. A stronger facilitation effect was found with a longer SOA compared to a shorter SOA for older participants, but not for younger participants. No such modulation patterns were found in the incongruence effect.

We found obvious gaze following and celebrity effects in older adults, suggesting that the cognitive ability to process celebrity status and gaze direction from face photos remains intact in older adults. Similar to younger individuals, both political leader and the star face enhanced gaze following in older adults. Previous studies have demonstrated that facial dominance, ethnic background, and social status influence gaze following in young adults (Capozzi et al., (Capozzi et al. 2016); Chen & Zhao, (Chen and Zhao 2015); Dalmaso et al., (Dalmaso et al. 2012), (Dalmaso et al. 2015); Jones et al., (Jones et al. 2010); Weisbuch et al., (Weisbuch et al. 2017); Zhang et al., (Zhang et al. 2021)), supporting the idea that top-down factors or social information can influence gaze following (Dalmaso et al., (Dalmaso et al. 2020); Jones et al., (Jones et al. 2010)). Our study extends these findings to older adults. More importantly, the influence of social information, such as the celebrity effect, on gaze following showed similar patterns in both older and younger adults. This may be due to an evolutionary tendency to follow and pay attention to important individuals within a group, such as political leaders or stars. Additionally, presenting three types of faces randomly within a block could enhance social comparison between the faces, consistent with the findings of Zhang et al. ((Zhang et al. 2021)), who reported that social comparison is activated when white and Asian faces are presented within the same block, thereby affecting gaze following (Zhang et al., (Zhang et al. 2021)). Future studies may investigate whether the celebrity effect observed in this study exists when faces are presented between blocks.

In quantifying gaze following, we analyzed three metrics: gaze cueing effect, facilitation, and incongruence effect, based on previous studies (Morillo-Mendez et al., (Morillo-Mendez et al. 2023); Slessor et al., (Slessor et al. 2016)). Older adults exhibited a larger facilitation effect under the longer SOA condition compared to the shorter SOA. Such SOA-related facilitation effect was not observed in young adults, suggesting a difference in the temporal course of gaze processing between older and younger adults. Older adults require longer processing times to effectively utilize gaze cues for task facilitation, consistent with previous findings (Deroche et al., (Deroche et al. 2016); Gayzur et al., (Gayzur et al. 2014)). These differences in temporal processing likely reflect cognitive slowing in older adults rather than a decline in social cognition. Additionally, our study used non-predictive gaze cues, as noted by McKay et al., where predictive gaze cues might show more significant age-related effects (McKay et al., (McKay et al. 2022)). Future research should explore whether the celebrity effect in gaze following persists with predictive gaze cues in older adults to better understand the potential differences in social cue processing across age groups.

In this study, we used more ecological gaze cues from real person photos to investigate the celebrity effect. As a result, we did not perfectly control for some factors, such as familiarity and individual attitudes of participants. Future studies could further explore the attitudes of elderly and young people towards political leaders and celebrity worship through the Implicit Association Test (IAT). The IAT can measure individual differences in implicit cognition (Greenwald et al., (Greenwald et al. 1998)), implicit personality traits, and emotional self-concepts such as anxiousness and anger (Schnabel et al., (Schnabel et al. 2006)). It has also been used to provide evidence for understanding ethnic identity in Africa (Lowes et al., (Lowes et al. 2015)). Future studies can utilize the IAT to investigate the celebrity effect on gaze following more thoroughly. Additionally, we used neural cues to further investigate the facilitation and inhibition effects, revealing that the impact of SOA on GCE in older adults is due to the benefits of facilitated orienting through gaze shifts rather than inhibitory processes. When studying gaze following in older adults, it is essential to account for the overall decline in processing speed. Therefore, quantifying gaze following should employ standardized metrics rather than simply subtracting uncued from cued conditions.

## Conclusion

These findings indicate that both older and young adults can integrate social information, such as celebrity identity, into their gaze following behavior. The celebrity effect on gaze following is consistent across both age groups, with stronger effects induced by the gazes of political leader and star compared to ordinary person. Additionally, a larger facilitation effect was observed under the longer SOA condition only in older adults. However, this facilitation effect was not present in young adults, highlighting an age-related difference in response to varying SOA conditions. Overall, the modulation of gaze following by celebrity identity was similar between older and younger adults.

## Data Availability

https://osf.io/s7zxu/

## Abbreviations

GCE: Gazing cue effect
SOA: Stimulus onset asynchrony
IAT: Implicit Association Test

## References

[CR1] Amenedo Losada, M. E., Lorenzo López, L., & Pazo Álvarez, P. (2012). *Response processing during visual search in normal aging: The need for more time to prevent cross talk between spatial attention and manual response selection*. 10.1016/j.biopsycho.2012.06.004 10.1016/j.biopsycho.2012.06.004 22743592

[CR2] Bennett, I. J., Golob, E. J., & Starr, A. (2004). Age-related differences in auditory event-related potentials during a cued attention task. *Clinical Neurophysiology,**115*(11), 2602–2615. 10.1016/j.clinph.2004.06.011 15465450 10.1016/j.clinph.2004.06.011

[CR3] Brooks, S. K. (2021). FANatics: Systematic literature review of factors associated with celebrity worship, and suggested directions for future research. *Current Psychology,**40*(2), 864–886. 10.1007/s12144-018-9978-4 10.1007/s12144-018-9978-4

[CR4] Capozzi, F., Becchio, C., Willemse, C., & Bayliss, A. P. (2016). Followers are not followed: observed group interactions modulate subsequent social attention. *Journal of Experimental Psychology: General,**145*(5), 531. 10.1037/xge0000167.supp 27031224 10.1037/xge0000167.supp

[CR5] Chen, Y., & Zhao, Y. (2015). Intergroup threat gates social attention in humans. *Biology letters,**11*(2), 20141055. 10.1098/rsbl.2014.1055 25716090 10.1098/rsbl.2014.1055 PMC4360112

[CR6] Ciardo, F., Marino, B. F., Actis-Grosso, R., Rossetti, A., & Ricciardelli, P. (2014). Face age modulates gaze following in young adults. *Scientific Reports,**4*(1), 4746. 10.1038/srep04746 24752250 10.1038/srep04746 PMC3994443

[CR7] Dalmaso, M., Castelli, L., & Galfano, G. (2020). Social modulators of gaze-mediated orienting of attention: A review. *Psychonomic Bulletin Review,**27*, 833–855. 10.3758/s13423-020-01730-x 32291650 10.3758/s13423-020-01730-x

[CR8] Dalmaso, M., Galfano, G., & Castelli, L. (2015). The impact of same-and other-race gaze distractors on the control of saccadic eye movements. *Perception,**44*(8–9), 1020–1028. 10.1177/0301006615594936 26562916 10.1177/0301006615594936

[CR9] Dalmaso, M., Galfano, G., Coricelli, C., & Castelli, L. (2014). Temporal dynamics underlying the modulation of social status on social attention. *PLoS One,**9*(3), e93139. 10.1371/journal.pone.0093139 24667700 10.1371/journal.pone.0093139 PMC3965511

[CR10] Dalmaso, M., Pavan, G., Castelli, L., & Galfano, G. (2012). Social status gates social attention in humans. *Biology Letters,**8*(3), 450–452. 10.1098/rsbl.2011.0881 22090207 10.1098/rsbl.2011.0881 PMC3367721

[CR11] Deroche, T., Castanier, C., Perrot, A., & Hartley, A. (2016). Joint attention is slowed in older adults. *Experimental Aging Research,**42*(2), 144–150. 10.1080/0361073X.2016.1132826 26890632 10.1080/0361073X.2016.1132826

[CR12] Driver, J., IV., Davis, G., Ricciardelli, P., Kidd, P., Maxwell, E., & Baron-Cohen, S. (1999). Gaze perception triggers reflexive visuospatial orienting. *Visual Cognition,**6*(5), 509–540. 10.1080/135062899394920 10.1080/135062899394920

[CR13] Faul, F., Erdfelder, E., Lang, A. G., & Buchner, A. (2007). G* Power 3: a flexible statistical power analysis program for the social, behavioral, and biomedical sciences. *Behavior Research Methods,**39*(2), 175–191. 10.3758/BF03193146 17695343 10.3758/BF03193146

[CR14] Fernandes, E. G., Tatler, B. W., Slessor, G., & Phillips, L. H. (2024). Age differences in gaze following: Older adults follow gaze more than younger adults when free-viewing scenes. *Experimental Aging Research,**50*(1), 84–101. 10.1080/0361073X.2022.2156760 36572660 10.1080/0361073X.2022.2156760

[CR15] Friesen, C. K., & Kingstone, A. (1998). The eyes have it! Reflexive orienting is triggered by nonpredictive gaze. *Psychonomic Bulletin, Review,**5*(3), 490–495. 10.3758/BF03208827 10.3758/BF03208827

[CR16] Friesen, C. K., Ristic, J., & Kingstone, A. (2004). Attentional effects of counterpredictive gaze and arrow cues. *Journal of Experimental Psychology: Human Perception and Performance,**30*(2), 319. 10.1037/0096-1523.30.2.319 15053691 10.1037/0096-1523.30.2.319

[CR17] Frischen, A., Bayliss, A. P., & Tipper, S. P. (2007). Gaze cueing of attention: visual attention, social cognition, and individual differences. *Psychological Bulletin,**133*(4), 694. 10.1038/s41598-023-37875-7 17592962 10.1038/s41598-023-37875-7 PMC1950440

[CR18] Gayzur, N. D., Langley, L. K., Kelland, C., Wyman, S. V., Saville, A. L., Ciernia, A. T., & Padmanabhan, G. (2014). Reflexive orienting in response to short-and long-duration gaze cues in young, young-old, and old-old adults. *Attention, Perception, Psychophysics,**76*, 407–419. 10.3758/s13414-013-0554-6 24170377 10.3758/s13414-013-0554-6 PMC3981892

[CR19] Greenwald, A. G., McGhee, D. E., & Schwartz, J. L. (1998). Measuring individual differences in implicit cognition: the implicit association test. *Journal of personality and social psychology,**74*(6), 1464. 10.1037/0022-3514.74.6.1464 9654756 10.1037/0022-3514.74.6.1464

[CR20] Hietanen, J. K., Nummenmaa, L., Nyman, M. J., Parkkola, R., & Hämäläinen, H. (2006). Automatic attention orienting by social and symbolic cues activates different neural networks: an fMRI study. *NeuroImage,**33*(1), 406–413. 10.1016/j.neuroimage.2006.06.048 16949306 10.1016/j.neuroimage.2006.06.048

[CR21] Ji, H., Yuan, T., Yu, Y., Wang, L., & Jiang, Y. (2022). Internal social attention: gaze cues stored in working memory trigger involuntary attentional orienting. *Psychological Science,**33*(9), 1532–1540. 10.1177/09567976221094628 35994624 10.1177/09567976221094628

[CR22] Jones, B. C., DeBruine, L. M., Main, J. C., Little, A. C., Welling, L. L., Feinberg, D. R., & Tiddeman, B. P. (2010). Facial cues of dominance modulate the short-term gaze-cuing effect in human observers. *Proceedings of the Royal Society B: Biological Sciences,**277*(1681), 617–624. 10.1098/rspb.2009.1575 10.1098/rspb.2009.1575 PMC2842686 19864283

[CR23] Langton, S. R., & Bruce, V. (1999). Reflexive visual orienting in response to the social attention of others. *Visual cognition,**6*(5), 541–567. 10.1080/135062899394939 10.1080/135062899394939

[CR24] Lien, M. C., Gemperle, A., & Ruthruff, E. (2011). Aging and involuntary attention capture: electrophysiological evidence for preserved attentional control with advanced age. *Psychology and aging,**26*(1), 188. 10.1037/a0021073 20973601 10.1037/a0021073

[CR25] Lowes, S., Nunn, N., Robinson, J. A., & Weigel, J. (2015). Understanding ethnic identity in africa: Evidence from the implicit association test (iat). *American Economic Review,**105*(5), 340–345. 10.1257/aer.p20151075 10.1257/aer.p20151075

[CR26] Madden, D. J., Parks, E. L., Davis, S. W., Diaz, M. T., Potter, G. G., Chou, Y. H., Chen, N. K., & Cabeza, R. (2014). Age mediation of frontoparietal activation during visual feature search. *NeuroImage,**102*, 262–274. 10.1016/j.neuroimage.2014.07.053 25102420 10.1016/j.neuroimage.2014.07.053 PMC4253678

[CR27] Mason, S. E. (1986). Age and gender as factors in facial recognition and identification. *Experimental Aging Research,**12*(3), 151–154. 10.1080/03610738608259453 3830234 10.1080/03610738608259453

[CR28] McKay, K. T., Grainger, S. A., Coundouris, S. P., Skorich, D. P., Phillips, L. H., & Henry, J. D. (2021). Visual attentional orienting by eye gaze: a meta-analytic review of the gaze-cueing effect. *Psychological Bulletin,**147*(12), 1269. 10.1037/bul0000353 35404635 10.1037/bul0000353

[CR29] McKay, K. T., Talipski, L. A., Grainger, S. A., Alister, M., & Henry, J. D. (2022). How does aging affect social attention? A test of competing theories using multilevel meta-analysis. *The Journals of Gerontology: Series B,**77*(8), 1454–1463. 10.1093/geronb/gbac052 10.1093/geronb/gbac052 PMC9371458 35279031

[CR30] Moore, C., Dunham, P. J., & Dunham, P. (2014). *Joint attention: Its origins and role in development*. Psychology Press.

[CR31] Morillo-Mendez, L., Mozos, O. M., & Schrooten, M. G. (2023). Gaze cueing in older and younger adults is elicited by a social robot seen from the back. *Cognitive Systems Research,**82*, 101149. 10.1016/j.cogsys.2023.101149 10.1016/j.cogsys.2023.101149

[CR32] Pavan, G., Dalmaso, M., Galfano, G., & Castelli, L. (2011). Racial group membership is associated to gaze-mediated orienting in Italy. *PLoS One,**6*(10), e25608. 10.1371/journal.pone.0025608 21991323 10.1371/journal.pone.0025608 PMC3186779

[CR33] Salthouse, T. A. (1996). The processing-speed theory of adult age differences in cognition. *Psychological review,**103*(3), 403. 10.1037/0033-295x.103.3.403 8759042 10.1037/0033-295x.103.3.403

[CR34] Schnabel, K., Banse, R., & Asendorpf, J. B. (2006). Assessment of implicit personality self-concept using the implicit association test (IAT): Concurrent assessment of anxiousness and angriness. *British Journal of Social Psychology,**45*(2), 373–396. 10.1348/014466605X49159 16762106 10.1348/014466605X49159

[CR35] Shi, J., Weng, X., He, S., & Jiang, Y. (2010). Biological motion cues trigger reflexive attentional orienting. *Cognition,**117*(3), 348–354. 10.1016/j.cognition.2010.09.001 20883983 10.1016/j.cognition.2010.09.001 PMC2967601

[CR36] Slessor, G., Laird, G., Phillips, L. H., Bull, R., & Filippou, D. (2010). Age-related differences in gaze following: Does the age of the face matter? *Journals of Gerontology Series B: Psychological Sciences and Social Sciences,**65*(5), 536–541. 10.1093/geronb/gbq038 20547536 10.1093/geronb/gbq038

[CR37] Slessor, G., Phillips, L. H., & Bull, R. (2008). Age-related declines in basic social perception: evidence from tasks assessing eye-gaze processing. *Psychology and aging,**23*(4), 812. 10.1037/a0014348. 19140652 10.1037/a0014348

[CR38] Slessor, G., Venturini, C., Bonny, E. J., Insch, P. M., Rokaszewicz, A., & Finnerty, A. N. (2016). Specificity of age-related differences in eye-gaze following: Evidence from social and nonsocial stimuli. *Journals of Gerontology Series B: Psychological Sciences and Social Sciences,**71*(1), 11–22. 10.1093/geronb/gbu088 25150512 10.1093/geronb/gbu088

[CR39] Wang, L., Wang, Y., Xu, Q., Liu, D., Ji, H., Yu, Y., Hu, Z., Yuan, P., & Jiang, Y. (2020). Heritability of reflexive social attention triggered by eye gaze and walking direction: Common and unique genetic underpinnings. *Psychological Medicine,**50*(3), 475–483. 10.1017/S003329171900031X 30829191 10.1017/S003329171900031X

[CR40] Weisbuch, M., Pauker, K., Adams, R. B., Jr., Lamer, S. A., & Ambady, N. (2017). Race, power, and reflexive gaze following. *Social Cognition,**35*(6), 619–638. 10.1521/soco.2017.35.6.619 10.1521/soco.2017.35.6.619

[CR41] Zhang, X., Dalmaso, M., Castelli, L., Fiorese, A., Lan, Y., Sun, B., Fu, S., & Galfano, G. (2021). Social attention across borders: A cross-cultural investigation of gaze cueing elicited by same-and other-ethnicity faces. *British Journal of Psychology,**112*(3), 741–762. 10.1111/bjop.12476 33010036 10.1111/bjop.12476

